# Relative variations of gut microbiota in disordered cholesterol metabolism caused by high‐cholesterol diet and host genetics

**DOI:** 10.1002/mbo3.491

**Published:** 2017-06-28

**Authors:** Tao Bo, Shanshan Shao, Dongming Wu, Shaona Niu, Jiajun Zhao, Ling Gao

**Affiliations:** ^1^ Scientific Center Shandong Provincial Hospital affiliated to Shandong University Jinan Shandong China; ^2^ Institute of Endocrinology Shandong Academy of Clinical Medicine Jinan Shandong China; ^3^ Department of Endocrinology and Metabolism Shandong Provincial Hospital affiliated to Shandong University Jinan Shandong China; ^4^ Department of Endocrinology Lin Yi People's Hospital affiliated to Shandong University Linyi Shandong China

**Keywords:** gut microbiota, high‐cholesterol diet, metabolism disorder

## Abstract

Recent studies performed provide mechanistic insight into effects of the microbiota on cholesterol metabolism, but less focus was given to how cholesterol impacts the gut microbiota. In this study, ApoE^−/−^ Sprague Dawley (SD) rats and their wild‐type counterparts (*n* = 12) were, respectively, allocated for two dietary condition groups (normal chow and high‐cholesterol diet). Total 16S rDNA of fecal samples were extracted and sequenced by high‐throughput sequencing to determine differences in microbiome composition. Data were collected and performed diversity analysis and phylogenetic analysis. The influence of cholesterol on gut microbiota was discussed by using cholesterol dietary treatment as exogenous cholesterol disorder factor and genetic modification as endogenous metabolic disorder factor. Relative microbial variations were compared to illustrate the causality and correlation of cholesterol and gut microbiota. It turned out comparing to genetically modified rats, exogenous cholesterol intake may play more effective role in changing gut microbiota profile, although the serum cholesterol level of genetically modified rats was even higher. Relative abundance of some representative species showed that the discrepancies due to dietary variation were more obvious, whereas some low abundance species changed because of genetic disorders. Our results partially demonstrated that gut microbiota are relatively more sensitive to dietary variation. Nevertheless, considering the important effect of bacteria in cholesterol metabolism, the influence to gut flora by “genetically caused cholesterol disorder” cannot be overlooked. Manipulation of gut microbiota might be an effective target for preventing cholesterol‐related metabolic disorders.

## 1 INTRODUCTION

Various kinds of bacteria, which densely colonized the gut of hosts, can be defined as gut microbiota (Costello et al., 2009). The diversity, structure, and stability of the gut microbiota can influence hosts’ nutrition, energy, metabolism, and immunity through intestinal nutrient‐sensing mechanisms, the gut–brain axis, or changes in intestinal permeability (Duca & Lam, 2014; Haro et al., 2016). Increasing evidence suggests that an imbalance of gut flora leads to metabolic and immunological diseases (Owyang & Wu, 2014). Roles that the gut microbiota play in high‐fat diet (HFD)‐induced insulin resistance and type II diabetes have been studied during the past few years, and the general relationship between the microbial community structure and obesity has been reported by several research groups (Mar Rodriguez et al., 2015; Rabot et al., 2010).

More recently, several studies have investigated the potential roles that gut microbiota play in regulating cholesterol metabolism and homeostasis in the body. It was found that bacteria in the colon are involved in both host cholesterol and bile acid (BA) metabolism pathways (Gerard, 2013). Cholesterol arriving at the large intestine should all be modified by colonic bacteria (Schoenheimer, 1931). Germ‐free rats only excreting unmodified cholesterol suggest that intestinal bacteria are responsible for cholesterol modification (Kellogg, 1974). Moreover, a small number of BAs, which escaped from the enterohepatic circulation (EHC), transited into the colon and was metabolized by the gut microbiota (Jonsson, Midtvedt, Norman, & Midtvedt, 1995). Despite the close relationship between the gut microbiota and cholesterol metabolism, studies focusing on the influence of single‐cholesterol factors on the gut flora are insufficient and the relationship is not clear enough (Martinez, Brown, & Walter, 2013).

Other than bacteria, the dysfunction of host genes may lead to an increased risk of metabolic syndrome (MS), such as hypercholesterolemia, and further atherosclerosis or cardiovascular disease. Apolipoprotein E (ApoE) is an important component of lipoproteins and is synthesized in the liver and distributed into several types of lipoproteins. It has been reported that ApoE participates in lipoprotein structure, stability, and lipid distribution. It also promotes the uptake and subsequent degradation of lipoproteins by binding to either the LDL receptor or the liver ApoE receptor (De Franca, Alves, & Hutz, 2004). ApoE plays important roles in the metabolism of lipoproteins, especially in the regulation of total cholesterol metabolism (De Franca et al., 2004; Schierwagen et al., 2015). ApoE^−/−^ mice have a phenotype of severe hypercholesterolemia and atherosclerosis regardless of diet (Zhang, Reddick, Piedrahita, & Maeda, 1992).

Gut microbiota participate in the metabolism of two classes of steroids: cholesterol originating from diet or synthesized de novo in the liver and the other from tissues, as well as BAs synthesized from cholesterol (Gerard, 2013). It was suggested that both diet variation and host metabolism can predispose animals to cholesterol disorders. However, it is unclear whether diet independently affecting bacteria, or cooperating with cholesterol metabolism. In other words, whether serum cholesterol works as the intermediate linkage between dietary variation and the microbiota profile remains of interest. It could also contribute to clarify the causality and correlation of the gut microbiota and endogenous/exogenous cholesterol disorders. In this study, ApoE^−/−^ Sprague Dawley (SD) rats were used as the model of endogenous cholesterol metabolic disorder. Rats fed a high‐cholesterol diet were considered the model for exogenous cholesterol disorders. The community structure and diversity of the gut microbiota were demonstrated by 16S rDNA high‐throughput sequencing. The relationship between the gut microbiota and endogenous/exogenous cholesterol disorders was discussed, based on the analysis.

## 2 MATERIALS AND METHODS

### 2.1 Animals

ApoE^−/−^ and wild‐type (WT) SD rats were purchased from Biocytogen (Beijing Biocytogen Co., Ltd., Beijing, China). Animals were raised in specific pathogen‐free (SPF) conditions under a 12‐hr dark–light cycle with free access to a normal chow diet (NC, commercial chow, 3.49 kcal/g; Keaoxieli Feed Co., Ltd., Beijing, China) and water. After 6 weeks of normal feeding, male WT and ApoE^−/−^ individuals were randomly separated into two dietary conditions and fed with NC and high‐cholesterol diet (HC, 98% commercial chow, 2% cholesterol, 3.14 kcal/g; Keaoxieli Feed Co., Ltd., Beijing, China), respectively (WT.NC, WT.HC, ApoE.NC, ApoE.HC). Each group contained three animals. Animals were individually housed. After 8 weeks of high cholesterol feeding, the levels of total serum cholesterol (TC), triglycerides (TG), LDL, and HDL were measured. Feces were collected once per day for 3 days. During the feces collection period, sterilized padding was daily changed. Each individual's feces from 3 days was mixed and then stored at −80°C for use.

Our research was approved by the Ethics Committee of Shandong Provincial Hospital. Procedures for all animal experiments were approved by Shandong Provincial Hospital Animal Care and Use Committee, and the methods were performed according to the approved guidelines.

### 2.2 DNA extraction and 16S marker amplification

Total microbial DNA from feces was extracted using the CTAB/SDS method. Briefly, 10% SDS was used as denaturant to break plasma membrane and release DNA. CTAB was added to denature the proteins and polysaccharides. DNA was then extracted by chloroform/isoamylol, precipitated by isopropanol, and washed by ethanol. The 16S rDNA V3‐V4 region was amplified by specific degenerate primers (341F: 5′‐CCTAYGGGRBGCASCAG‐3′; 806R: 5′‐GGACTACNNGGGTATCTAAT‐3′) with unique barcodes. PCR products were detected by electrophoresis using 2% agarose gels, and samples in the range of 400–450 bp were excised and extracted using a gel extraction kit (GeneJET, Thermo Scientific) for further analysis.

### 2.3 High‐throughput sequencing

16S rDNA libraries were generated by a NEB Next^®^ Ultra^™^ DNA Library Prep Kit for Illumina (New England Biolabs, USA). Libraries were qualified on the Qubit@2.0 Fluorometer (Thermo Scientific) and Agilent Bioanalyzer 2100 system. The eligible libraries were sequenced on an Illumina Hiseq platform. Paired end reads of 250 bp were generated. High‐throughput sequencing was performed using the Illumina Hiseq platform Novo gene (Novo gene Bioinformation Technology. Beijing, China).

### 2.4 Data analysis

Overlapping paired end reads from reads 1 and 2 of the original DNA fragments (Raw Data) were merged. Next, the reads were assigned to each sample using the unique barcodes. Sequences (Clean Data) were analyzed using the Quantitative Insights into Microbial Ecology (QIIME, Version 1.7.0) software package, and in‐house Perl scripts were used to analyze alpha diversity and beta diversity. Reads were filtered by QIIME quality filters. Sequences with ≥97% similarity were assigned to the same optimal taxonomic units (OTUs). Then, a representative sequence was chosen for each OTU to annotate the taxonomic information of that unit. All OTUs were subsequently analyzed for abundance and diversity. For alpha diversity analysis, rarefaction curves and rank abundance curves were generated by R project (Version 2.15.3). Intergroup differences in alpha diversity were analyzed by a nonparametric test. For beta diversity, QIIME (Version 1.7.0) was used to calculate unweighted pair group method with arithmetic mean (UPGMA). R project was chosen to calculate principal component analysis (PCA) and principal coordinates analysis (PCoA). Intergroup differences in beta diversity were analyzed by a nonparametric test. *T*‐test and Wilcox test were chosen for analysis of two groups; Tukey's and Wilcox's tests were chosen for analysis between more than two groups.

### 2.5 Statistical analysis

The differences between the relative species abundance of multiple groups were compared using nonparametric tests, followed by a multiple comparison test for subgroups by LSD (least significant difference). All statistical analyses were performed using SPSS version 18.0 for Windows (Chicago, IL, USA).

## 3 RESULTS

### 3.1 Dietary variation and genetic manipulation changed the serum cholesterol profiles in rats

**Figure 1:**
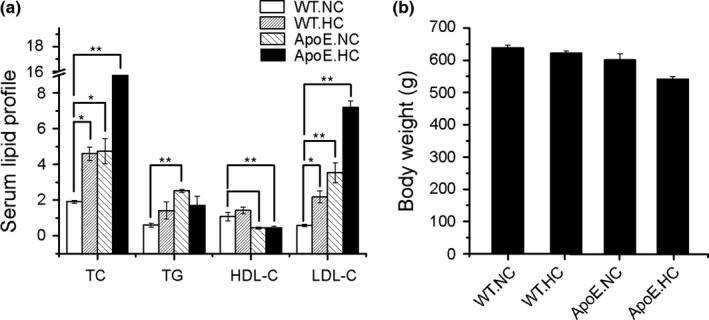
Phenotypes of different groups with different genotypes and dietary conditions. (a) The serum TC, TG, LDL‐C, HDL‐C levels of different groups; (b) body weight of different groups. The differences among the four groups were compared using nonparametric tests, *p <* *.05 versus WT.NC group. **p <* *.01 versus WT.NC group. Error bars are calculated as a standard error (SEM)

Compared to the WT.NC group, levels of serum TC and LDL‐C in the other three groups were statistically different. The high‐cholesterol diet treatment of the ApoE.HC group obviously increased this discrepancy. Body weight of the ApoE.HC group was slightly decreased, but with no significant difference (Figure 1).

### 3.2 Dietary variation is more effective than genetic manipulation in changing microbial structure (alpha diversity)

**Figure 2:**
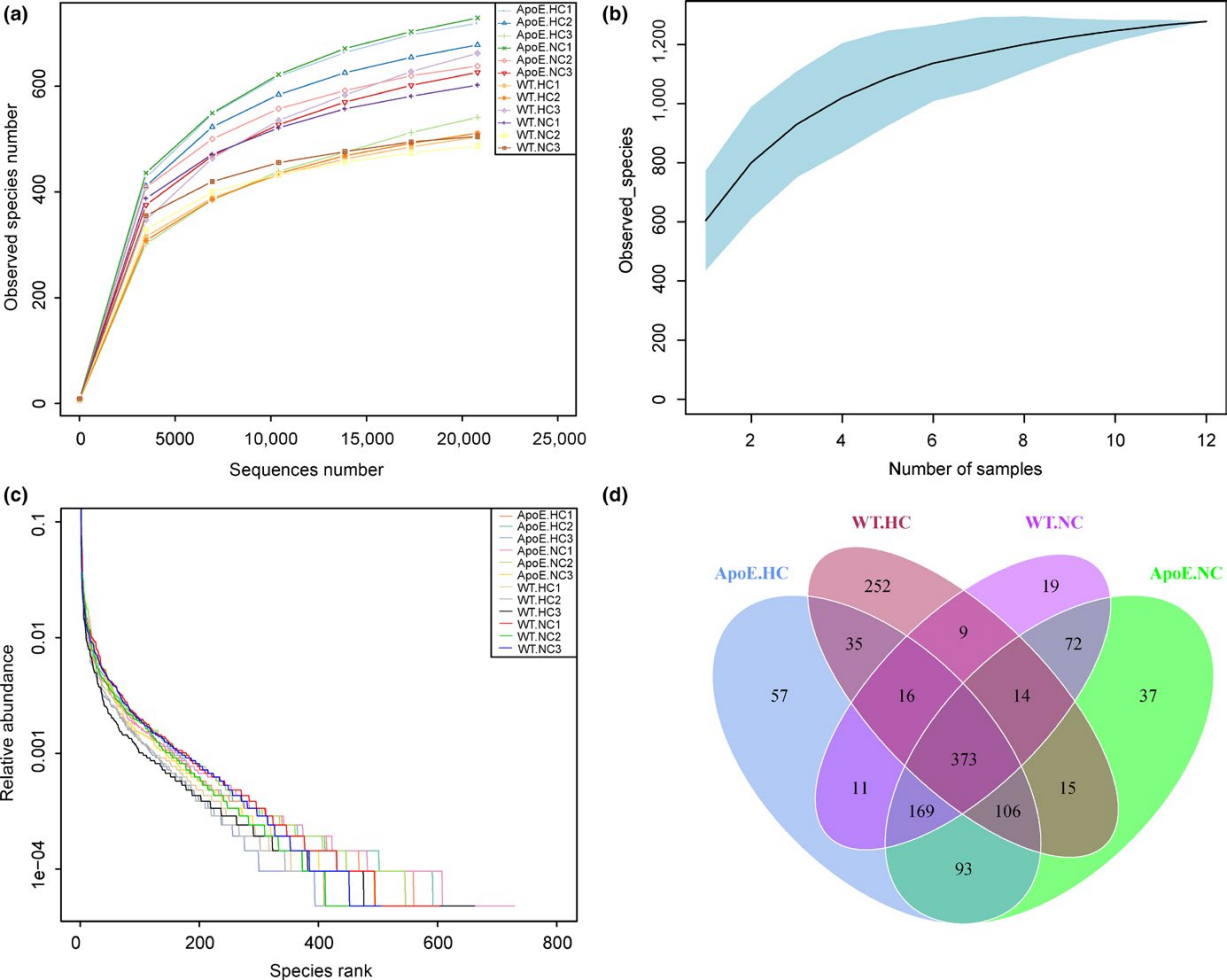
Alpha diversity analysis of different groups. (a) Rarefaction curve is generated by setting the number of sequence as *x*‐axis, and the number of observed species as *y*‐axis. The curve reflected the relationship between the quantity of observed species and sequences. The “plateaued” shape of the curve indicated that enough samples/sequences were obtained to cover the majority of species. (b) Species accumulation curve is generated by setting sample number as *x*‐axis, and the number of observed species as *y*‐axis. Species accumulation curves described the number of species along with the increase in sample size. (c) Rank abundance curve is generated by setting relative abundance of OTU as *y*‐axis, and OTU number as *x*‐axis. The spanning of the curve in *x*‐axis reflects the richness of the species does the sample has, while the smooth reflects the evenness of the species. (d) Each circle in the Venn diagram represented one group noted by the name of same color. The numbers located in the overlapping area represented the number of OTUs share with respective groups. The numbers located in the individual area represented the number of OTUs peculiar to the representative group

**Figure 3:**
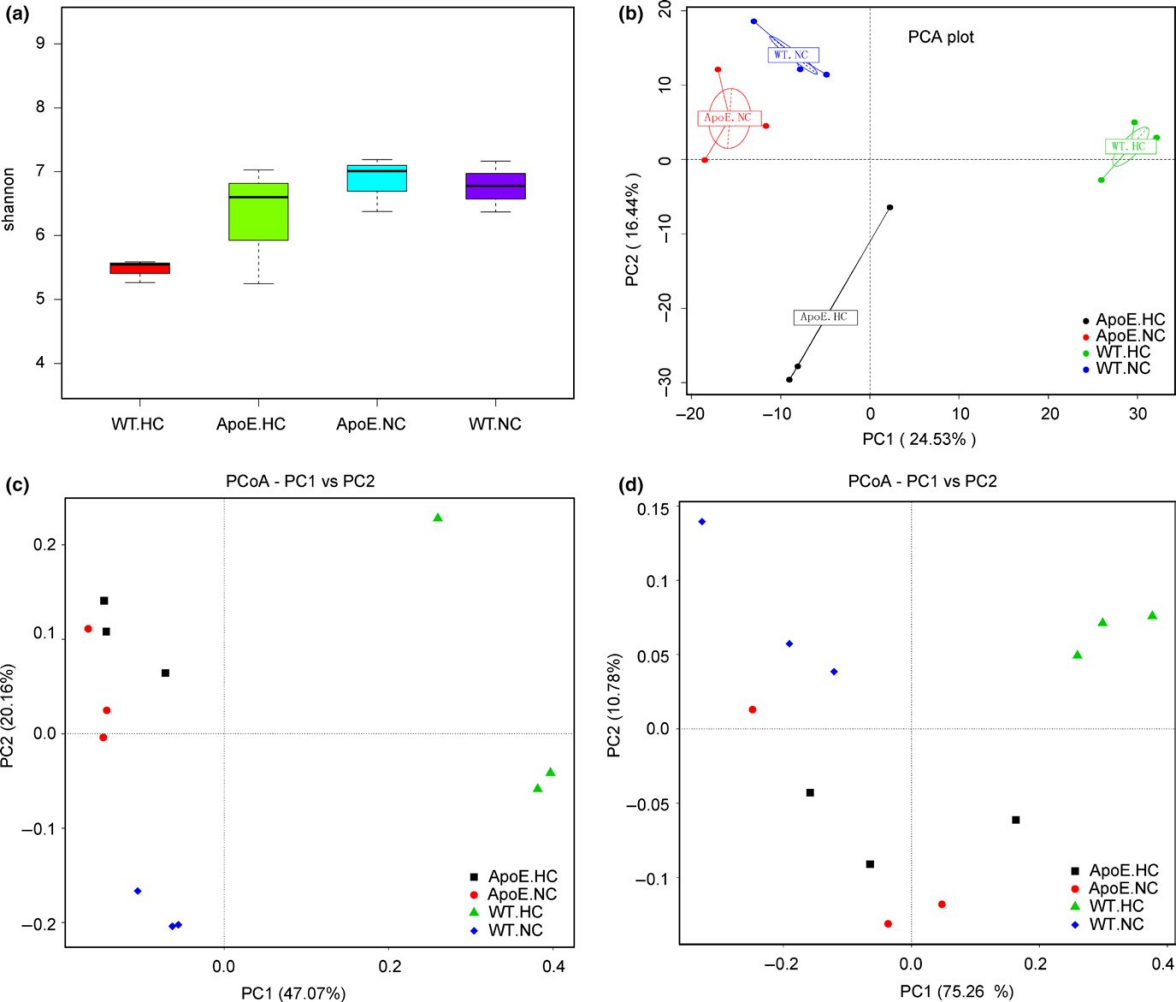
Beta diversity analysis of different groups. (a) Shannon index box plot; (b) PCA; (c) and (d) unweighted unifrac PCoA and weighted unifrac PCoA. PC1 and PC2 in *x*‐ and *y*‐axis represented two principle discrepancy components between groups, and the percentage in bracket means contribution value to the discrepancies by the component. Dots represent samples. Samples in same group share same color

Illumina Hiseq high‐throughput sequencing was performed to evaluate the bacteria abundance. The overall information of sample reads before and after merging and quality filtering is listed in Table 1. On average, we obtained 74,106 total reads. Reads were then merged based on the overlapped sequence to generate tags. On average, 43,765 total tags were obtained. We clustered the tags using a 97% similarity cutoff to obtain an average of 665 OTUs. Species accumulation curves, rarefaction curves, and rank abundance curves were analyzed together to test the sufficiency of the sequence collection (Figure 2). In species accumulation/rarefaction curves, the curve of the observed species number plateaued with the increase in samples/sequences number, which indicated that enough samples/sequences were obtained to cover the majority of species. The rank abundance curve can be used to evaluate the experimental stability. Venn diagram was generated based on the OTU classification. There was obvious deviation of the WT.HC group from the other three groups (with 252 OTUs in particular). The two WT groups shared 412 OTUs in common, with proportions of 50.2% for WT.HC and 60.3% for WT.NC. However, the similarity was more obvious between two ApoE groups, with 741 OTUs in common, and the percentage shared was 86.2% for ApoE.HC, and 84.3% for ApoE.NC. From the OTU distributions presented in the Venn diagram, it seemed that the deviations caused by a high‐cholesterol diet within the WT groups were reduced in ApoE groups. The species diversity of each group was assessed by a box plot of the Shannon index, as shown in Figure 3a. The results suggested that a high‐cholesterol diet could reduce the diversity of the gut microbiota, as shown by the Shannon index of the WT.HC group (*p *=* *.048). To deeply illustrate the differences in gut flora between these groups, beta‐diversity analysis was performed to analyze the phylogenetic relationship and relative abundance of species.

**Table 1:** Preprocessing statistics and quality control of raw data

Sample	Raw PE	Combined	Qualified	Nochime	Base(nt)	AvgLen(nt)	Q20	Q30	GC%	Effective%
WT.HC1	112,686	74,636	58,070	53,705	22,441,106	418	97.83	95.82	53.08	47.66
WT.HC2	125,696	87,146	70,102	65,229	27,186,382	417	98.01	96.14	53.07	51.89
WT.HC3	128,027	93,394	75,344	71,305	29,806,307	418	97.98	96.05	53.25	55.7
ApoE.HC1	66,088	55,971	45,407	41,187	17,013,764	413	97.41	94.67	53.61	62.32
ApoE.HC2	59,997	51,180	41,798	38,389	15,874,786	414	97.41	94.65	53.54	63.98
ApoE.HC3	39,352	32,998	26,225	24,247	10,090,831	416	97.21	94.3	52.93	61.62
ApoE.NC1	59,774	50,078	40,219	36,799	15,262,714	415	97.32	94.45	53.8	61.56
ApoE.NC2	54,155	46,402	38,601	36,084	14,839,308	411	97.37	94.56	53.92	66.63
ApoE.NC3	66,983	56,422	45,291	42,529	17,651,419	415	97.39	94.61	53.54	63.49
WT.NC1	66,030	57,124	47,178	42,911	17,695,203	412	97.35	94.54	53.48	64.99
WT.NC2	56,622	48,315	39,596	37,219	15,336,510	412	97.34	94.53	53.14	65.73
WT.NC3	53,865	45,826	37,778	35,580	14,639,562	411	97.35	94.52	53.44	66.05
Average	74,106	58,291	47,134	43,765	18,153,158	414	97	95	53	61

Raw PE, original data derived from the indicated primer pair; Combined, original data were assembled by overlapped sequence; Qualified, low‐grade quality and short‐length reads were filtered out to generate qualified data; Nochime, chimera of qualified sequences were filtered out to generate effective sequences; Base, the base number of total data; AvgLen, average length of qualified tags; Q20, the percentage of bases with sequencing error rate <1%; Q30, the percentage of bases with sequencing error rate <.1%; GC%, GC content; Effective%, the percentage of effective tags.

### 3.3 Dietary variation is more effective than genetic manipulation in changing intergroup discrepancies (beta diversity) of gut microbiota

Unconstrained ordination was performed to describe the discrepancies between the groups. PCA was developed on the basis of primary species classification and the abundance of samples. It can estimate the main discrepancies, in the OTUs, between groups using the distance of sample dots. Then, PCoA was calculated based on samples’ distance matrices, which were generated based on their group species phylogenic and evolutionary relationships. In our study, PCA showed that the species discrepancy was obvious between the four groups, especially for WT.HC. The WT.NC and ApoE.NC groups were the most similar pair, which suggested that genetics were not as important as dietary variation for species modification (Figure 3b). However, aside from the same results for the WT.HC group, PCoA, especially weighted unifrac PCoA (considering the quantity of species during phylogenetic calculating), showed decreased differences between the other three groups when compared with PCA measurements. This meant that although the species were different between those three groups, their “phylogenetic types,” which can be reflected by PCoA, were more difficult to clearly classify (Figure 3c and d). These results indicated that HC diet caused apparent variation in gut flora, whereas genetic manipulation was not only less effective but also reduced the discrepancy.

**Figure 4:**
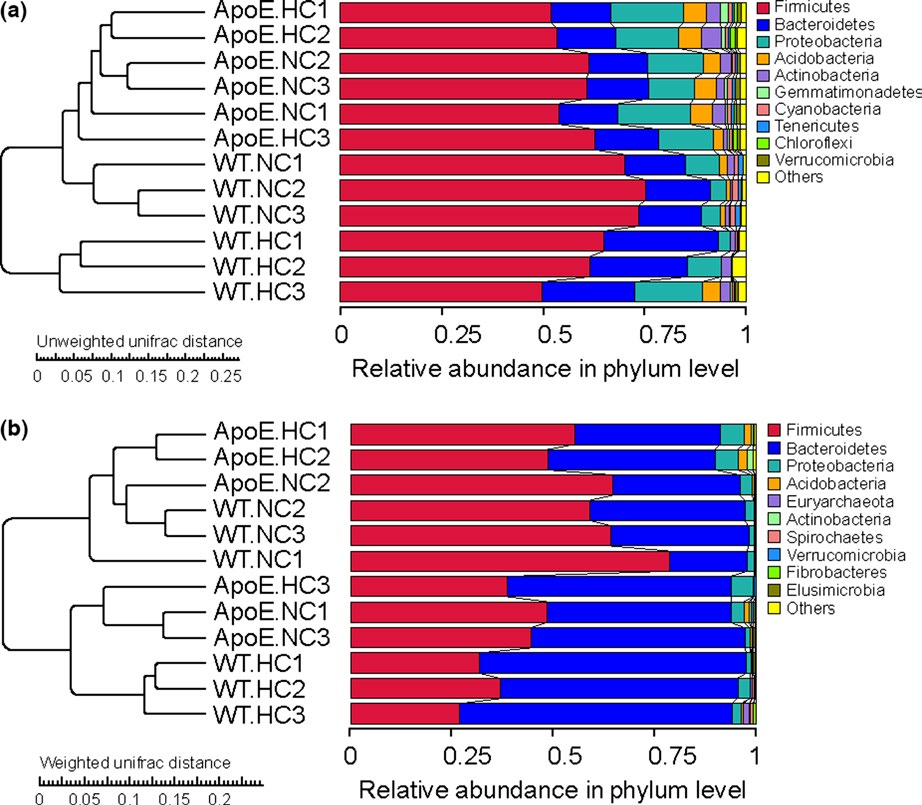
UPGMA analysis of different groups. (a) UPGMA clustering tree based on unweighted unifrac distance. (b) UPGMA clustering tree based on weighted unifrac distance. Left side of the diagram is the structure of clustering tree. Right side is the relative abundance of different phylum. The difference between unweighted unifrac and weighted unifrac is that the former only including the factor of species classification and evolutionary relationship, while the latter bringing the species abundance factor into calculation

We next estimated the differences using the UPGMA, which was generated using the perspective of cluster analysis, by constructing clustering trees on the basis of the group's phylogenetic data (Highton, 1993). The results are shown in Figure 4. In our results, two types of algorithms both indicated that the phylogenetic relationship of the WT‐HC group was relatively far from the other three groups, especially in the unweighted unifrac analysis (Figure 4a). The other three groups were relatively clearly separated in the unweighted unifrac analysis, whereas the differences were diminished in the weighted unifrac analysis, as demonstrated by the node sites and the length of branches (Figure 4b).

### 3.4 Both dietary and gene variation could change the relative abundance of some metabolic representative species

**Figure 5:**
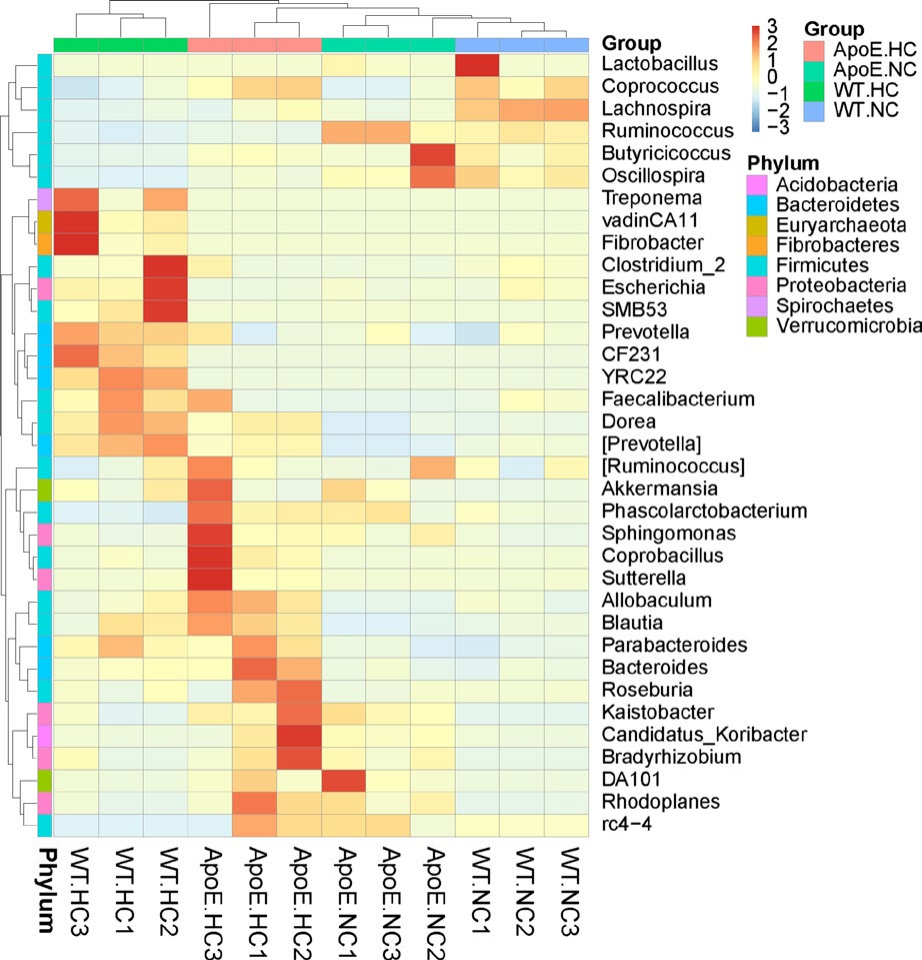
The heat map of relative abundance of different phylum. Samples information is transverse listed, and species annotations are longitudinal shown. The left clustering tree is species‐related clustering tree, and the upper tree is sample‐related clustering tree. The heat map was performed by discrepancies of species‐relative abundance between samples, with colors gradually changed from deep red to deep blue, in accordance with high relative abundance to low. The data were “Z” value, which were calculated based on standardized relative abundance

**Figure 6:**
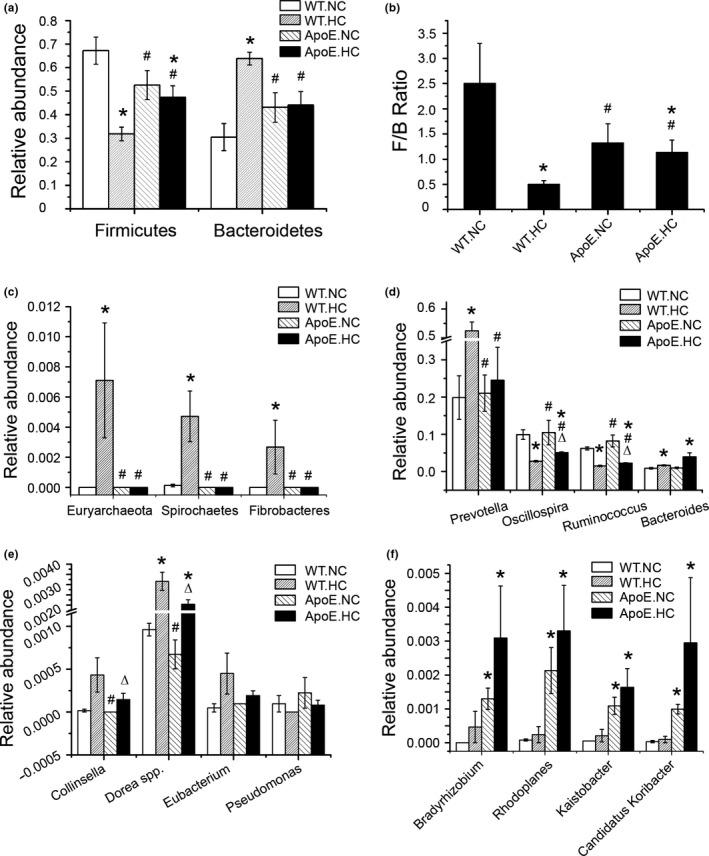
Relative abundance analysis of some metabolic representative species. (a) Relative abundance of Firmucutes and Bacteroidetes of different groups; (b) F/B ratio of different groups; (c) relative abundance of the phylum of Euryarchaeota, Spirochaetes, Fibrobacteres of different groups; (d) relative abundance of the genus of *Prevotella*,* Oscillospira*,* Ruminococcus*,* Bacteriodes* of different groups; (e) relative abundance of some low abundance species with apparent differences derived from dietary conditions; (f) relative abundance of some low abundance species with apparent differences in ApoE groups. The differences among groups were compared using nonparametric tests. *p <* *.05 versus respective species in WT.NC group; #*p *<* *.05 versus respective species in WT.HC group; △*p *<* *.05 versus respective species in ApoE.NC group. Error bars are calculated as a standard error (SEM)

The relative abundance of bacteria was analyzed at the level of phylum, class, order, family, and genus. The structure and stability of the gut microenvironment can be denoted by the relative abundance of different types of bacteria. The variation in the abundance of representative organisms was summarized on a heat map and column diagram, shown in Figures 5 and 6. The heat map was generated using the relative abundance of different phylum (Figure 5). Obvious discrepancies appeared in HC groups compared with NC groups, as shown by the samples’ clustering tree on the upper right of the heat map diagram.

We then continued to analyze the abundance of representative species in detail (Figure 6). First, we found that the relative abundance of the phylum Firmicutes and Bacteroidetes obviously changed between the four groups (Fig 6a). The ratio of the phylum Firmicutes to Bacteroidetes (F/B) was analyzed using nonparametric tests (Fig 6b). The F/B ratio of the WT.HC group was decreased compared to the other three groups, suggesting that HC diet remarkably influenced the major components of intestinal microbiota. A decreased F/B ratio in the ApoE groups occurred, but was not obvious (no statistical difference), and the dietary influence within the ApoE groups was not as obvious as it was in the WT groups. It seemed dietary variation played more of an effect on the gut microbiota than genetic manipulation.

Other remarkable distinctions occurred in the Euryarchaeota, Spirochaetes, and Fibrobacteres phyla, which were hardly detected in the other three groups, as well as the genus *Prevotella*, which showed obvious increase in the WT.HC group (Figure 6c and d). *Prevotella*, which mostly colonizes in obese individuals, contains members involved in carbohydrate and amino acid fermenting, as well as acetate and hydrogen producers (Vargas‐Bello‐perez, Cancino‐Padilla, Romero, & Garnsworthy, 2016). *Euryarchaeota* is the main archaeal species that colonized the intestine (Horz & Conrads, 2010). These methanogens may harm health through interspecies hydrogen transfer or generating toxic volatile methylated derivatives (Conway de Macario & Macario, 2009; Michalke et al., 2008). The major members of the phylum *Spirochaetes* detected in the WT.HC group belonged to the OTU of *Treponema* (90.4% of the phylum), which contains pathogens of the physiological flora (Antal, Lukehart, & Meheus, 2002).

Some species also showed some interesting variations influenced by dietary condition. The genera *Ruminococcus* and *Oscillospira*, influenced by the dietary condition, both were found decreased in the HC groups (Figure 6d). Moreover, variations in the low abundance species cannot be overlooked, as they may have specific metabolic functions that have been reported to be important (Ondov, Bergman, & Phillippy, 2011). Of these low abundance species, the genus *Collinsella* was obviously increased in the WT.HC group. *Dorea* spp., which belongs to *Clostridium*, showed an increased likelihood for abundance in HC groups, although it was not the predominant species in the community. Moreover, the relative abundance of the genera *Eubacterium* and *Pseudomonas* (Figure 6e) were slightly heightened in both HC groups, so was the genus *Bacteroides* (Figure 6d), which showed a much higher population, especially in the ApoE.HC group. Based on those results, it seemed that dietary cholesterol levels played more effective roles in modifying the structure of gut microbiota than genetic manipulation. However, there were still some exceptions. *Kaistobacter*, in the order of Sphingomonadales, Candidatus Koribacter of Acidobacteriales, and some nitrogen‐fixing bacteria, *Bradyrhizobium* and *Rhodoplanes* of Rhizobiales, were obviously increased in ApoE groups, which showed less relative with feeding condition. The aforementioned species together composed 1.48% of the average relative abundance in ApoE groups, which cannot be overlooked (Figure 6f).

## 4 DISCUSSION

Gut microbiota are closely associated with host metabolism and nutrition. A variety of factors influence the stability of the gut microbiota: diet, pathogens, antibiotics, probiotics, and prebiotics, which can be associated with the development of MS (Cani et al., 2007; Economopoulos et al., 2016; Isolauri, Rautava, Collado, & Salminen, 2015; Turnbaugh et al., 2006; Zhang et al., 2014; Zhao, 2013). Dietary condition is an important factor that changes the structure of gut microbiota. The causative role of diet on the gut flora has been globally recognized (Zhang et al., 2010, 2012). Gut microbiota also closely relate to cholesterol uptake and metabolism. The problem is that recent studies in animal models performed mechanistic insight into the effects of the microbiota on metabolic pathologies, but less focus was given to how these pathologies impact the gut microbiota. Previous studies have focused on the role that the gut microbiota play on the metabolic effects of cholesterol and BA, by using Koch's postulates as considering whole gut microbiota in its entirety (Gerard, 2013; Sayin et al., 2013; Zhong et al., 2015). Hu et al. reported that a high‐cholesterol diet changed the gut microbiota profile in rats (Hu et al., 2015), although the major point of this study was still discussing the causative role of microbiota in cholesterol metabolism. Recently, a few studies have begun to describe the influence of cholesterol on the gut microbiota. It was reported that the addition of plant sterol esters in the diet could alter the gut microbial profile (Martinez, Perdicaro, et al., 2013). In another study, plasma HDL‐C levels were positively associated with the abundance of Bacteroidetes in human fecal samples (Martinez, Lattimer et al., 2013). Thus, it is important to understand how the host phenotype affects the microbiome, including through diets, altered cholesterol excretion, or changes in the BA pool.

In our study, high cholesterol feeding led to a decreased ratio of F/B, and these results were similar to those reported in previous studies focusing on gut microbiota profiles and metabolic diseases (Hu et al., 2015; Zhu et al., 2013). Compared to the WT.HC group, the influence of genetic manipulation on the F/B ratio was not obvious, although there was a relatively higher serum cholesterol level. These results can partially demonstrate that dietary factors play an important role in maintaining gut structure and stability. A similar variation tendency was observed for the relative abundance of several phylum or species, including the genera *Prevotella, Ruminococcus*, and *Oscillospira*, and the phyla Euryarchaeota and Spirochaetes. *Prevotella* is involved in the fermentation of carbohydrates and amino acids and is also a producer of acetate and hydrogen (Vargas‐Bello‐perez et al., 2016). These H_2_‐producing components were often detected in combination with a relatively high abundance of *Archaea*, which are H_2_‐oxidizing methanogenic organisms, for example, Euryarchaeota. Methanogens in Euryarchaeota can provide syntrophic support for some types of bacteria involved in fermentation through interspecies hydrogen transfer. These fermentation bacteria could be at least opportunistic pathogens in gut flora (Conway de Macario & Macario, 2009). Moreover, the methanogenic members of Euryarchaeota can effectively transform heavy metals or metalloids into volatile methylated derivatives which may be more toxic than the original compounds (Michalke et al., 2008). It seems that an increased abundance of Euryarchaeota, together with pathogen members, may be harmful to one's health. *Ruminococcus* was considered as a cell collaborator in digestive absorption of carbohydrate and polysaccharide, which may promote people to be overweight or suffer from obesity due to excessive energy storage. Some low abundance species also showed correlation with dietary variation. The Coriobacteriaceae family had been linked to host lipid metabolism in animal and human studies (Claus et al., 2011; Martinez et al., 2009). It was reported that atherosclerosis in humans was associated with an increase in *Collinsella*. (Karlsson et al., 2012). *Dorea* spp. showed an increased abundance only in the WT.HC group. This result was partially consistent with the findings by Lahti et al. (2013) who found a positive correlation between bacteria related to *Dorea* spp. and TC and LDL‐C. The genera *Eubacterium*,* Pseudomonas*, and *Bacteroides* were slightly increased in both of the HC groups. These organisms are involved in cholesterol and BA metabolism, including cholesterol reduction and BA deconjugation. BA sulfatase activity has been detected in intestinal isolates belonging to *Pseudomonas*, which also are recognized as pathogens (Gerard, 2013).

Our results showed that dietary variation may play more effective roles in changing the gut flora compared to metabolism disorders caused by genetic manipulation. This phenomenon was more or less similar with Zhang's research on microbiota discrepancy between HFD versus ApoA‐I defect model (Zhang et al., 2010), which indicated that a high‐fat diet had a dominating role in shaping the gut microbiota despite a complete host genome. Although our results had few exceptions, *Kaistobacter* in the order of Sphingomonadales, Candidatus Koribacter of Acidobacteriales, and some nitrogen‐fixing bacteria, *Bradyrhizobium* and *Rhodoplanes* in the order of Rhizobiales, were obviously increased in two ApoE groups, which showed weaker relation with regard to the feeding condition. Acidobacteria are newly classified phylum of bacteria. Because they have only recently been discovered and the large majority had yet to be cultured, the metabolism of these bacteria is not well understood (Barns, Cain, Sommerville, & Kuske, 2007; Quaiser et al., 2003). It is reported that Rhizobiales can change their gene expression profile to cope with BAs or with antimicrobial activities.

In our results, it was interesting that the microbial discrepancy derived from dietary change in the WT groups was obviously reduced in ApoE groups, which indicated that the high‐cholesterol level caused by a genetic mutation may lower the influence of diet. It had been well established that BA metabolism is under tight control to prevent potential toxicity and ensure appropriate cholesterol catabolism. The host cholesterol level may not remarkably influence the quantity of BA which arrives in the colon. As a consequence, BA metabolism‐related species might be less affected. Another possible reason may be the level of food intake. It was reported that compared to WT counterparts, ApoA‐I‐deficient animals had decreased food intake, but were much more obese and had higher levels of blood glucose (Zhang et al., 2010). In our study, an average weight loss of the ApoE group (WT group, 573.83 ± 23.07; ApoE group, 515.67 ± 16.42; *p *<* *.05) may suggest that the energy intake was not equal to the WT groups. Therefore, the stability of BA metabolism and varied energy intake may be a restricting factor that diminishes the effect of the high‐cholesterol diet on the gut microbiota.

Our study suggested that dietary variation might play more effective roles in changing gut flora compared to the metabolic disorder caused by genetic manipulation. In this sense, we thought that the change of microbial profile due to dietary intervention might be uncorrelated with host cholesterol metabolism. Nevertheless, considering the more important effect of bacteria on cholesterol metabolism than in lipid metabolism, the influence on gut flora by a “genetically caused cholesterol disorder” cannot be overlooked. Further analysis should be performed to illustrate the detailed causative role of diet on the gut microbiota.

## CONFLICT OF INTEREST

None declared.
